# Concurrent production and relative quantification of vasicinone from in vivo and in vitro plant parts of Malabar nut (*Adhatoda vasica* Nees)

**DOI:** 10.1007/s13205-017-0882-7

**Published:** 2017-08-16

**Authors:** Jitendriya Panigrahi, Saikat Gantait, Illa C. Patel

**Affiliations:** 1 Department of Biotechnology, Shri A. N. Patel P. G. Institute, Anand, Gujarat 388001 India; 2 All India Coordinated Research Project on Groundnut, Directorate of Research, Bidhan Chandra Krishi Viswavidyalaya, Kalyani, Nadia, West Bengal 741235 India; 3 Department of Genetics and Plant Breeding, Faculty of Agriculture, Bidhan Chandra Krishi Viswavidyalaya, Mohanpur, Nadia, West Bengal 741252 India; 4 Department of Life Sciences, Hemchandracharya North Gujarat University, Patan, Gujarat 384265 India

**Keywords:** *Adhatoda vasica*, HPTLC, Malabar nut, Medicinal plant, Vasicinone

## Abstract

The present study documents a simultaneous production and comparative assessment of extracted vasicinone from in vivo (leaves and stems) and in vitro (leaves, stems and calli) plant parts of *Adhatoda vasica* Nees, a well-known medicinal plant. High-performance thin layer chromatography (HPTLC) analysis of the above-mentioned plant parts, collected at their 60-day-old growth stage, was performed via methanolic extraction and with the aid of toluene:butanol:butyl acetate (9:0.5:0.5; *v/v/v*) solvent system. The method was validated with the help of aluminium sheet precoated with silica gel 60 F_254_ TLC plates, following the ICH guidelines in order to maintain accuracy, precision and repeatability. Correlation coefficient, limit of detection and limit of quantification values were found to be reasonable. The outcome revealed a linearity that ranged between 2 and 6 µg/spot. During the comparison of estimated vasicinone quantity from in vivo and in vitro plant parts, it was evident that in vitro samples produced relatively higher vasicinone than that of the in vivo counterparts. Maximum vasicinone (6.402 ± 0.010% of dry weight) production was quantified from in vitro leaves followed by calli (5.222 ± 0.092% of dry weight) and in vitro stems (2.007 ± 0.041% of dry weight). On the other hand, in vivo leaves and stems produced comparatively lower quantities of vasicinone (2.412 ± 0.139 and 1.933 ± 0.046% of dry weight, respectively) suggesting the in vitro clonal propagation as a superior approach in comparison to in vivo propagation. Nonetheless, simultaneous production from both the sources (in vivo and in vitro plant parts) provides a new avenue for augmented production of vasicinone.

## Introduction

*Adhatoda vasica* Nees (syn, *Justicia adhatoda* L.), commonly known as Malabar Nut (or Vasaka) is a perennial shrub and belongs to the family Acanthaceae. It grows in sub-Himalayan tracts and has conventionally been utilized in Ayurvedic and Unani medicine for more than 2000 years (Jayapaul et al. 2005). It is basically an evergreen shrub of 1–2.5 m height with opposite ascending branches producing a vile smell and bitter taste. Several ethnopharmacological studies on *A. vasica* reported the aerial portions of the plant (like stem, leaf, flower, fruit and seeds) to contain vasicine, vasicinone (Suthar et al. 2009), vasicine acetate and 2-acetyl benzyl amine (Ignacimuthus and Shanmugam 2010), adhatodine and vasicoline (Ahmad et al. 2009). *A. vasica* leaves and stems have been utilized as a herbal medication for allergen-induced respiratory diseases, rheumatism, malarial fever, gastrointestinal disorders, haemorrhage, skin diseases and many other ailments, occurring in countries like India, Nepal, Pakistan and Sri Lanka (reviewed by Claeson et al. 2000). *A. vasica* has been included in the WHO manual for health workers in Southeast Asia owing to its recurrent usage, conveniently devoid of any serious side effects (World Health Organization 1990). Vasicinone, the central autooxidation by-product of vasicine, has been documented to exhibit bronchodilatory, cardiac-stimulating and anti-anaphylactic effects (Bhide et al. 1976; Shinawie 2002). One of the commercial produces of *A. vasica* is Adulsa, a cough syrup (Roja et al. 2011). There is a significant requirement of *A. vasica*-based drugs in the Indian subcontinent and this demand is being satisfied from the natural plant population so far. Inaptly, seed germination rate of *A. vasica* is poor and traditional propagation through vegetative approach is inadequate (Mathew et al. 1998), which prompts significant depletion of the source of pharmaceutically important metabolites like vasicinone. This existing scenario calls for an alternative approach in the form of in vitro propagation that could complement the traditional system of propagation, and crop up as a better source for secondary metabolites extraction. Nowadays plant organ, tissue and cell culture system have been exhaustively explored as a substitute for the production of secondary metabolites, avoiding any loss in natural plant population in majority of the medicinal plant species (Ramachandra Rao and Ravishankar 2002; Komaraiah et al. 2003; Gantait et al. 2011). Yet, the comparative assessment of secondary metabolite among in vitro and in vivo plant parts is of utmost importance from an economic viewpoint; however, such study has been attempted only once (Garg et al. 2016) in *A. vasica*, to the best of our knowledge. The propagation conditions (in vitro or in vivo) assisting the accumulation of high concentration of vasicinone in *A. vasica* are undetermined as well.

Earlier reported techniques for the vasicinone quantification comprises high-performance capillary electrophoresis (HPCE) (Avula et al. 2008), reversed-phase high-performance liquid chromatography (RPHPLC) (Garg et al. 2016), and ultra-high-performance liquid chromatography coupled with triple quadrupole linear ion trap mass spectrometry (UHPLC–ESI/MS/MS) (Singh et al. 2016), along with high-performance thin layer chromatography (HPTLC) as well. Presently, HPTLC is being widely used for its systematic method validation, consistency in the quantification of analytes (precisely in nanogram levels) and cost effectiveness. The prime benefit of HPTLC is curtailment of time and cost per analysis (Dharmender et al. 2010). Effective use of HPTLC has been well documented in some major economically important medicinal plants for their respective secondary metabolite assay (Gantait et al. 2011; Swain et al. 2012; Varghese et al. 2013; Bala et al. 2015; Kumar et al. 2016; Shuayprom et al. 2016), but only three reports are available on vasicinone quantification from in vivo plant samples of *A. vasica* (Das et al. 2005; Suthar et al. 2009; Roja et al. 2011) with none on comparative quantification (between in vivo and in vitro plant parts) of vasicinone.

The present study was therefore focused on the simultaneous production and relative assessment of vasicinone through HPTLC from both the in vivo and in vitro plant parts of *A. vasica*, and optimizing the major sink of accumulation.

## Materials and methods

### Collection of plant materials

**Fig. 1:**
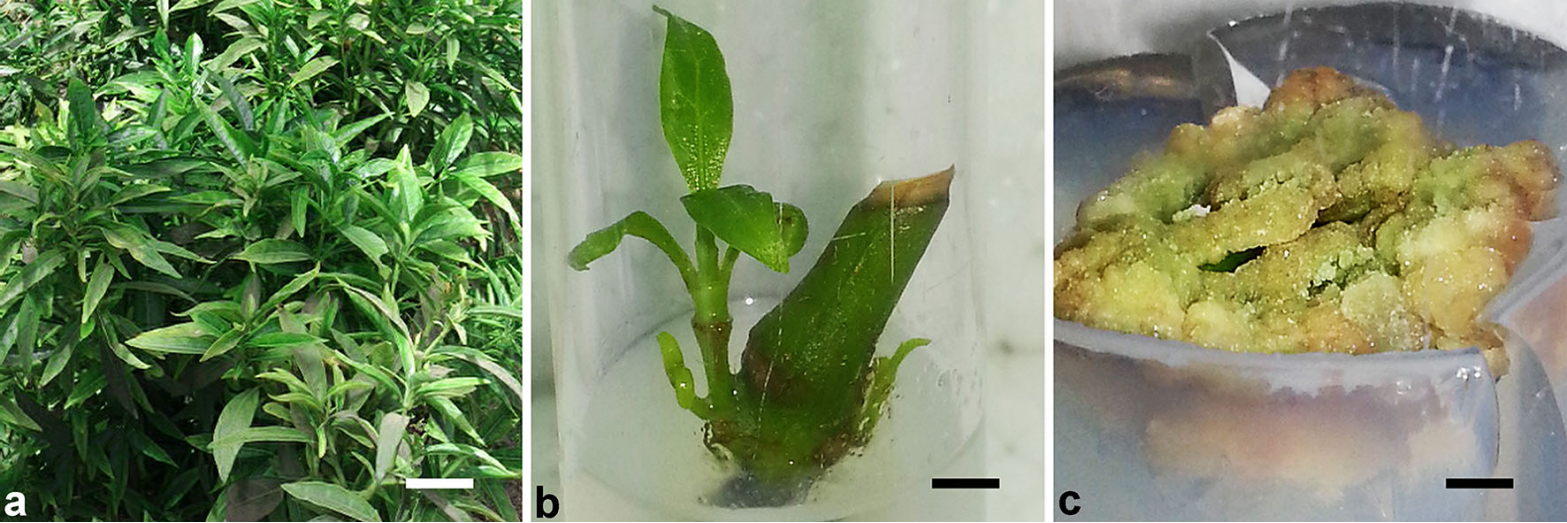
In vivo and in vitro propagation of *Adhatoda vasica* Nees for simultaneous production, extraction and quantification of vasicinone. **a** In vivo propagation through stem-cuttings; **b** in vitro multiple shoot and leaf regeneration from nodal-segment explant; **c** in vitro callus induction from nodal-segment explant

The stem-cuttings of *A. vasica* Nees were collected from Medicinal and Aromatic Research Centre, Anand Agriculture University, Anand, Gujarat, India, and propagated through traditional vegetative method (Fig. 1a). The freshly developed and fully opened 60-day-old leaves along with tender stems of similar growth stage were harvested to measure the vasicinone content.

The in vivo-collected nodal-segment explants were inoculated in Murashige and Skoog (1962) (MS) medium fortified with 1.1 mg/l of *N* ^6^-benzyladenine for in vitro initiation and multiplication of axillary shoots following the protocol of Panigrahi and Patel (2014). Simultaneously, for in vitro callus induction, the in vivo-collected nodal segments were inoculated in MS medium fortified with 1 mg/l of 2,4-dichlorophenoxy acetic acid. Leaves and stems of in vivo plants as well as in vitro-regenerated leaves, stems (Fig. 1b) and induced calli (Fig. 1c) were used for HPTLC analysis considering vasicinone as standard.

### Preparation of sample solution

To develop the appropriate fingerprinting along with effective extract preparation, sample solutions were evaluated. The in vivo and in vitro samples (weighing 1 g of the oven dried and powdered) were extracted using methanol (50 ml) under reflux in a thimble with extractor volume 200 and 300-ml flask volume of Soxhlet apparatus (Borosil^®^, Mumbai, India). The extracts were filtered across Whatman filter paper (no. 1) and concentrated under vacuum and subsequently the volume was adjusted with methanol to develop a concentration of 1 mg/ml that were used for the chromatography analysis. One milligramme standard of vasicinone (purity: 97%, Natural Remedies Pvt. Ltd, Bengaluru, India) was dissolved in 1 ml of methanol. It was further diluted to get the concentration of 0.01 mg/ml. Toluene:butanol:butyl acetate (9:0.5:0.5; *v/v/v*) was used for co-chromatography with the different extracted samples.

### Chromatographic analysis

Five-microlitre solutions from each sample were spotted in the form of bands by 100-µl syringe (from Hamilton^®^, Sigma-Aldrich, USA) in a CAMAG Linomat 5 Automatic Sample Spotter (Camag Muttenz, Switzerland) on an aluminium sheet precoated with silica gel 60 F_254_ TLC plates (20 × 10 cm with 0.25 mm thickness) (E. Merck, Germany). After drying, the plate was developed in toluene:butanol:butyl acetate (9:0.5:0.5; *v/v/v*) solvent system to a distance of 8 cm and scanned at 233 nm. The peak areas were measured and the quantity of vasicinone was estimated using the calibration curve with the assistance of winCATS Planar Chromatography Manager software. The whole procedure was carried out at 25 ± 2 °C with a relative humidity of 40% while the analytical grade chemicals used in the experiment were purchased from Loba chemicals, Mumbai, India.

### Method validation

The HPTLC method for quantification of vasicinone was validated for the following parameters—linearity, calibration curve, instrumental precision and repeatability. Calibration curve was drawn up and tested for linearity as well as validation of the suggested method. The coefficient of variance (CV%), correlation coefficient (*r*), and the linearity of results were analysed. Calibration curve of each sample was obtained by plotting peak areas vs concentrations of applied samples. The instrumental precision and the repeatability of the scanning technique was confirmed by repetitive scanning (*n* = 9) of the same spot (on TLC plate) of each sample and expressed as relative standard deviation (%RSD).

*Sensitivity* The sensitivity of the proposed method was deduced against limit of detection (LOD) and limit of quantification (LOQ). The LOD and LOQ were calculated based on the gradient of calibration (S) curve and standard deviation (sdv) of the blank sample following the formula proposed by Alam et al. (2011).

*Specificity* It was established by evaluating standard vasicinone, test samples and diluent. The bands for vasicinone from test sample solutions were affirmed via correlating the *R*_f_ and spectra of the bands to those of the standard vasicinone. Analysis of peak purity of all the compounds was performed analogizing the spectra at start, middle, and end positions of the bands. The *R*_f_ values and colour of the resolved bands were noted.

### Data analysis

The HPTLC analysis for the quantitative determination of vasicinone on different in vivo and in vitro plant samples was replicated thrice and the recorded data were put to one-way analysis of variance. The significant variation among the vasicinone quantity (mean ± SE) was analysed by Duncan’s multiple range test (Duncan 1955) at *P* = 0.05 level with the aid of SPSS (Version 20, SPSS Inc. Chicago, USA) software package.

## Results and discussion

**Fig. 2:**
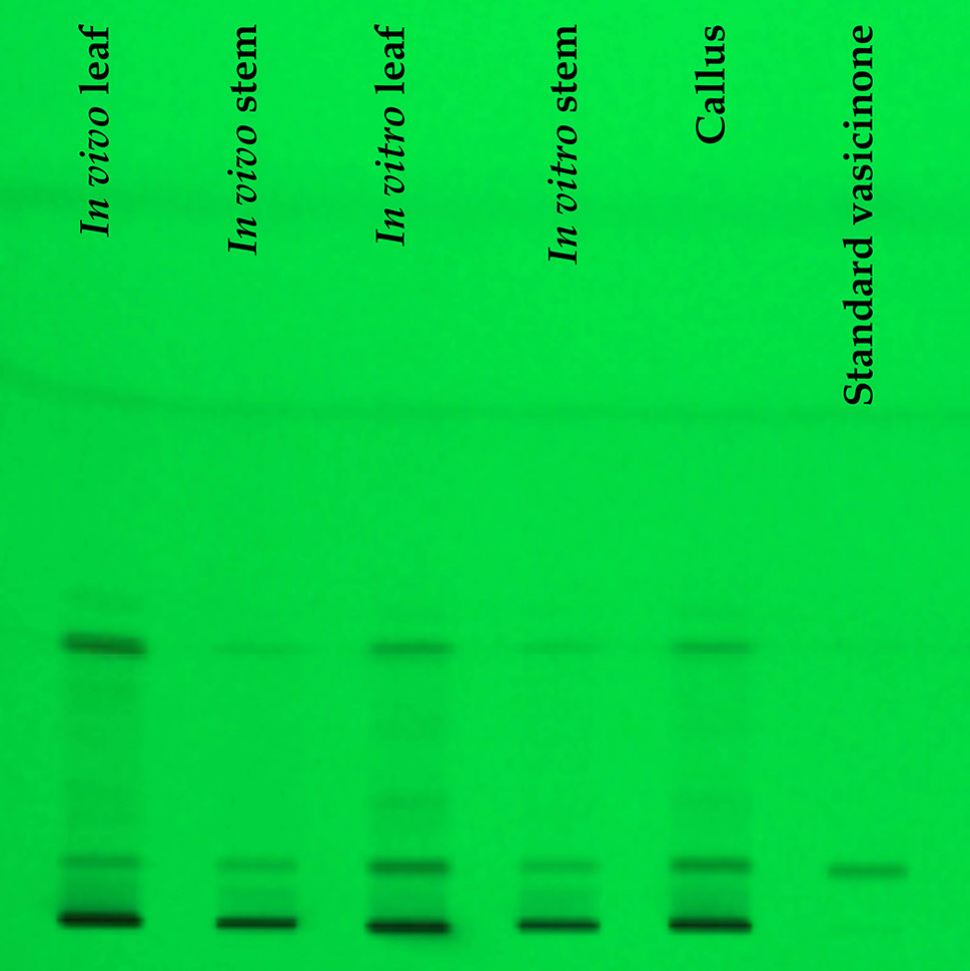
TLC profile of the *Adhatoda vasica* Nees samples at 233 nm remission; samples extracted from in vivo and in vitro sources along with vasicinone as standard

**Fig. 3:**
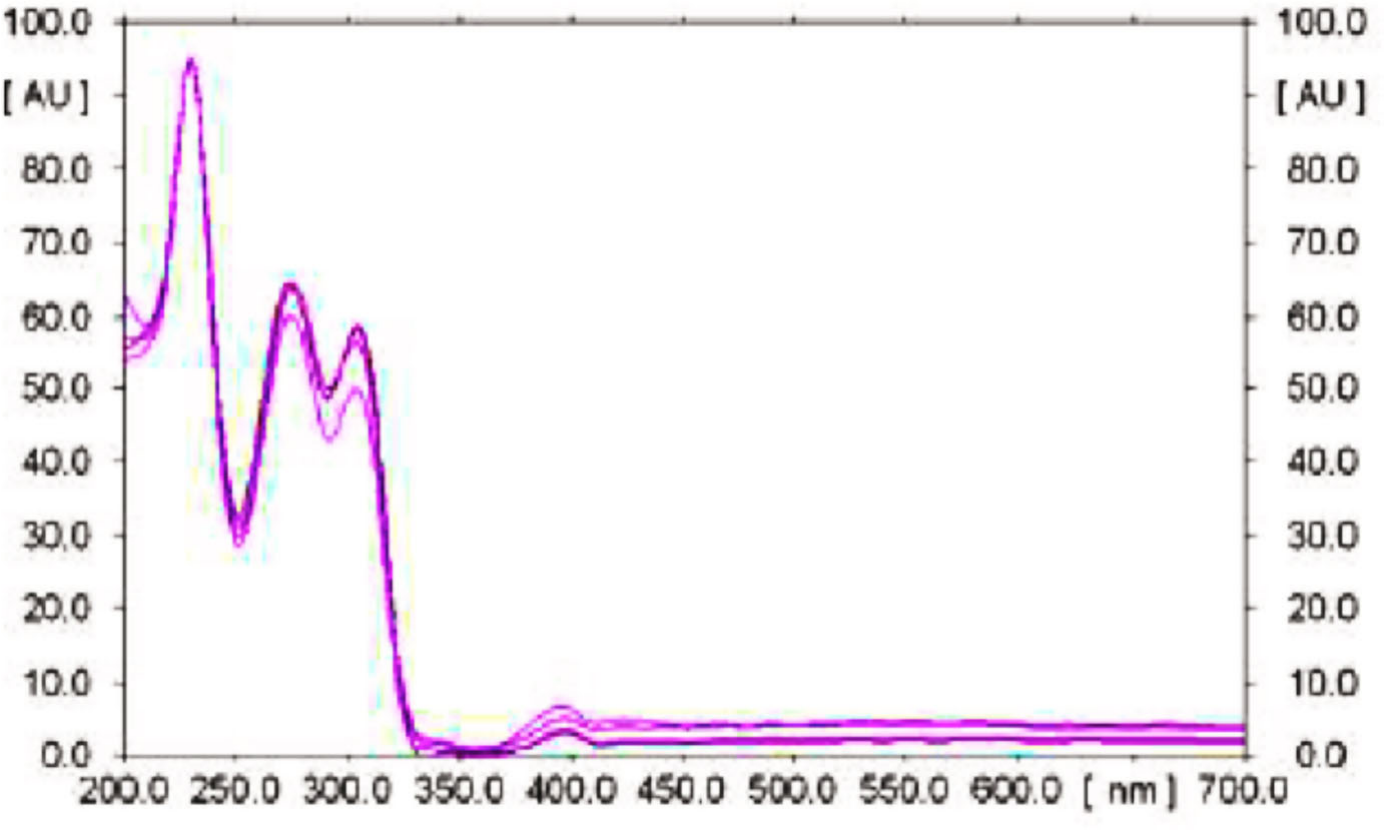
Overlay spectra of methanolic extract of different parts of in vivo and in vitro grown *Adhatoda vasica* Nees at 233 nm

**Fig. 4:**
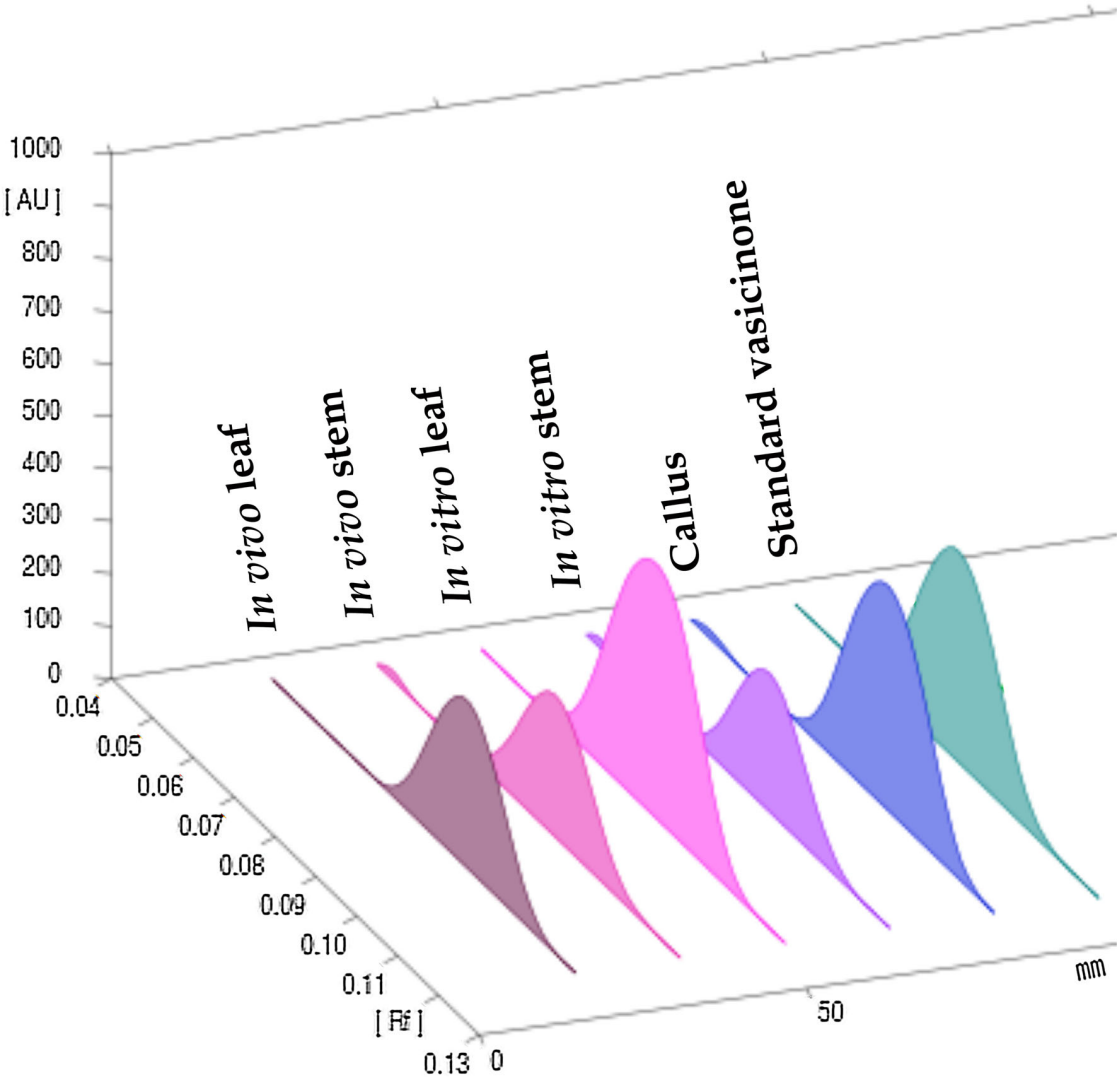
HPTLC fingerprinting (all tracks at 233 nm) of vasicinone along with the methanolic extract of different parts of in vivo and in vitro grown *Adhatoda vasica* Nees

Preparatory TLC studies showed that the toluene:butanol:butyl acetate (9:0.5:0.5; *v/v/v*) solvent system was ideal as a mobile phase that produced *R*_f_ 0.4–0.09 for vasicinone and well-resolved spots were found from the in vivo and in vitro plant samples (Fig. 2). The spots were observed at 233 nm and the resultant three-dimensional densitogram configurations of the test samples and standard vasicinone demonstrated that the peaks for all the samples corresponding to *R*_f_ 0.04 were overlaying (Figs. 3, 4). The features of the corresponding spectrum of this peak were noted to match precisely with each other, revealing the compounds analogous to *R*_f_ of the standards and the in vivo and in vitro plant samples to be identical.

### Analytical method validation

**Fig. 5:**
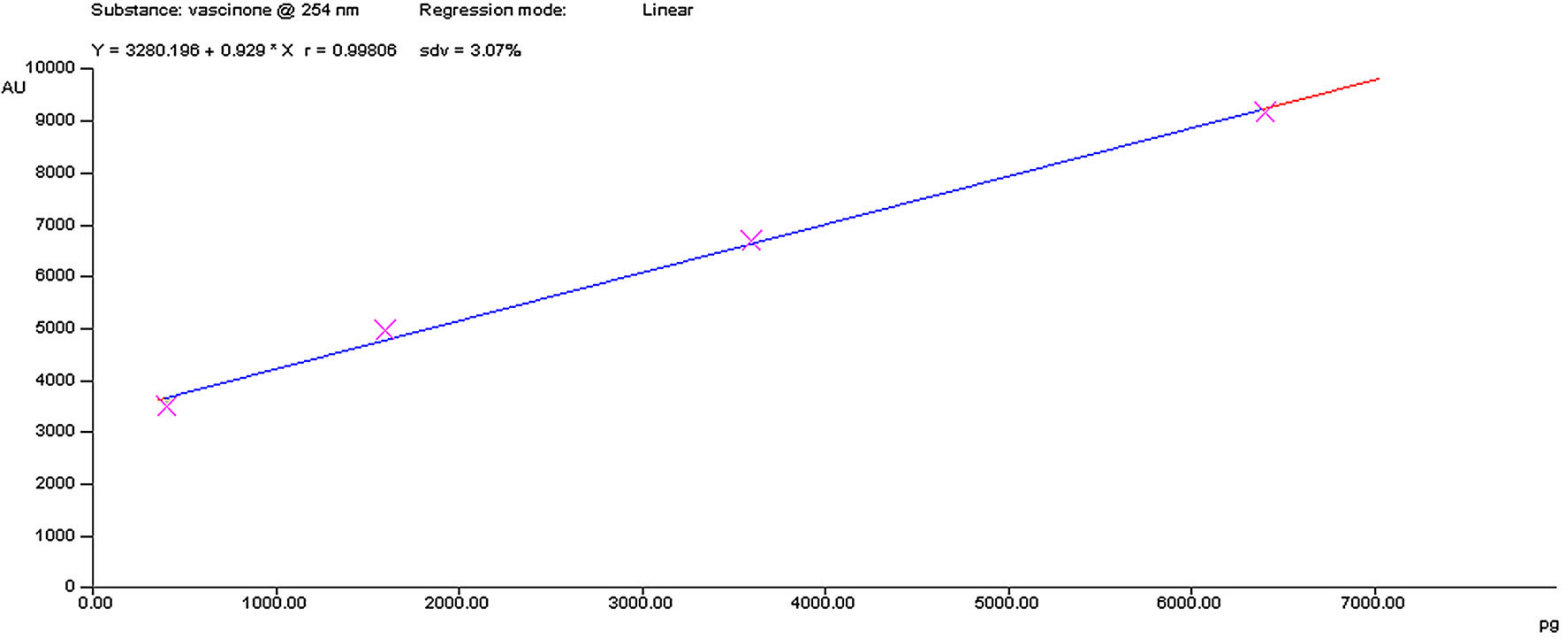
Calibration curve of vasicinone

The linearity of the calibration curve was found between 2 and 6 µg/spot with a respective correlation coefficient (*r* value of 0.99 with standard deviation—sdv of 3.07), for vasicinone (Fig. 5). The regression equation for the calibration plot for vasicinone is *Y* = 1235 + 754.4 × *X*. Percentage (*w/w*) of vasicinone was deduced using the peak area factor.

The densitometric method was found to be precise with RSD 20.93% (for intra-day) and 19.96% (for inter-day) by scanning the same spot of vasicinone simultaneously, with a %CV value of 1.884 (intra-day) and 1.797 (inter-day), respectively. The repeatability of the method was investigated by evaluating nine applications of the same standard solution %CV = 1.884 for intra-day and 1.797 for inter-day (*n* = 9). Validation data determines the precision of the methodology adapted in the current study in agreement with the 1 µg/spot LOD and 3 µg/spot LOQ.

**Fig. 6:**
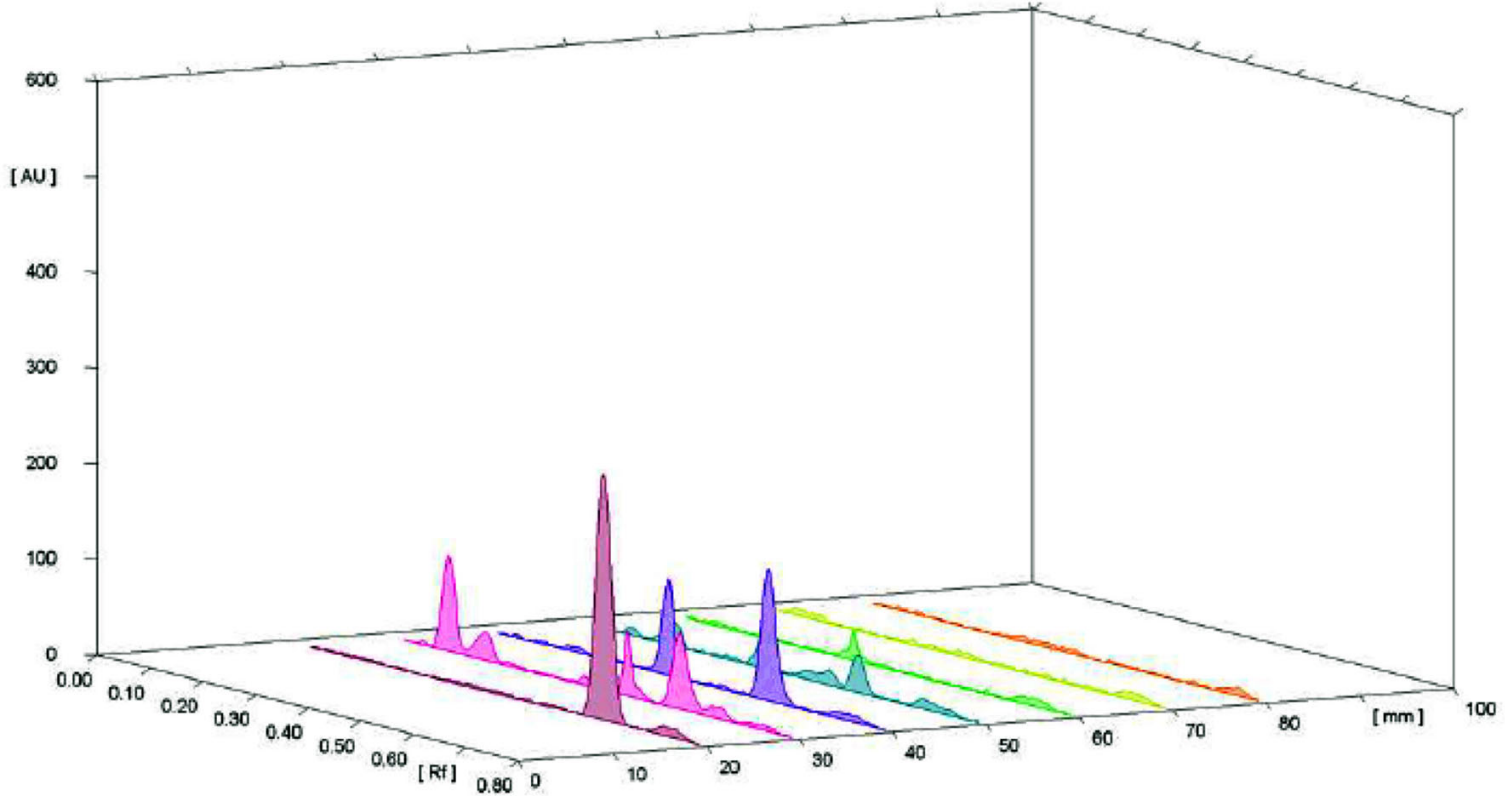
Specificity in *Adhatoda vasica* Nees samples at 233 nm remission

For the instruments precision, the specificity study was carried out taking vasicinone as standard, wherein test samples along with the mobile phase and diluent were taken on different tracks. The method proved to be specific for vasicinone since it resolved the compound (*R*_f_ = 0.04) well in the presence of other components (Fig. 6).

### Determination and comparative analysis of vasicinone content from in vivo and in vitro plant samples

**Fig. 7:**
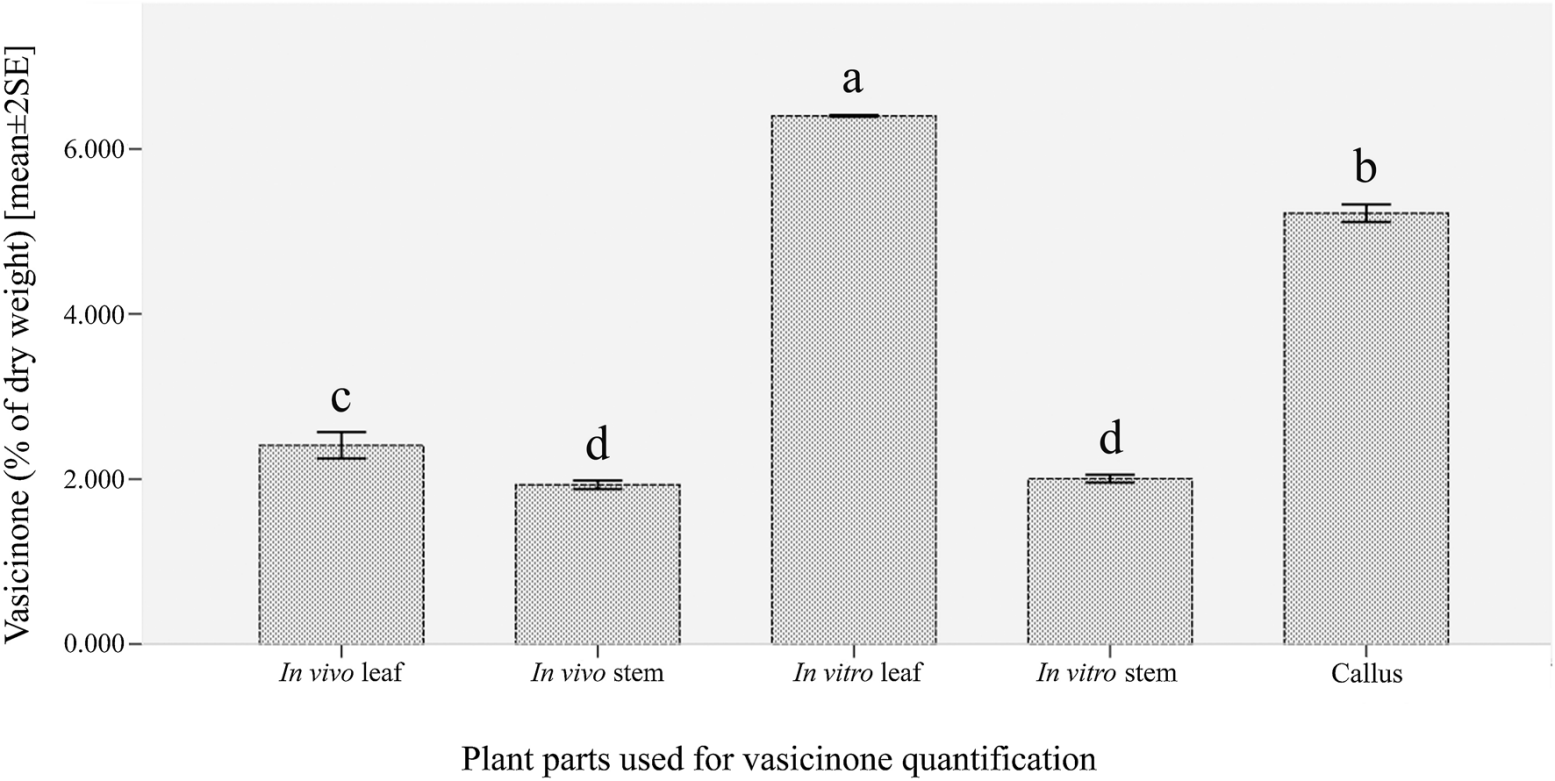
Comparative vasicinone content (mean value ± standard error bar; *n* = 3) in various (in vivo and in vitro) plant parts of *Adhatoda vasica* Nees. Columns with the *different letters* are significant at *P* = 0.05 based on one-way analysis of variance followed by Duncan’s multiple range test (Duncan 1955)

 The quantities (mean ± SE) of vasicinone procured from various in vivo and in vitro plant samples, expressed as % of dry weight, are given in Fig. 7. It was evident from our study that vasicinone could be produced concurrently from different parts of the plant irrespective of in vitro or in vivo environments. Such outcome certainly will encourage the pharmaceutical industry to use both these methods to complement each other to meet the bulk demand of *A. vasica*-based drug industries in the Indian subcontinent for vasicinone source. However, in a comparison, during in vivo and in vitro vasicinone production, it was detected that samples from in vitro condition produced a better yield than the other one. The highest quantity of vasicinone (6.402 ± 0.010% of dry weight) was measured from in vitro leaves followed by calli (5.222 ± 0.092%) and in vitro stems (2.007 ± 0.041%). In contrast, in vivo leaves and stems produced comparatively lower levels of vasicinone (2.412 ± 0.139 and 1.933 ± 0.046%, respectively) (Fig. 7). This result is corroborated by the previous report of Roja et al. (2011) who found 3–4% vasicinone in natural plant sample and 5–6% vasicinone in tissue cultured plant sample, when assessed through HPLC. It was noteworthy to mention that the vasicinone quantity from the in vitro leaves was almost 2.5-fold higher than its in vivo counterpart. An earlier study (Das et al. 2005) reported that the vasicinone production in leaves (in vivo) was higher (0.047%; *w/w*) in comparison to stem (in vivo) (0.027%; *w/w*) of *A. vasica*, which supports our results where we found leaf samples to be the superior choice for vasicinone extraction than the other plant parts. Significant variation in vasicinone content was observed among in vitro leaf, callus and in vivo leaf; nonetheless, in vitro stem and in vivo stems yielded comparable quantities of vasicinone. The coefficient of variation (CV%) of individual samples was recorded to be 8.133% (in vivo leaf), 3.35% (in vivo stem), 0.219% (in vitro leaf), 0.129% (in vitro stem) or 2.864% (callus). The quantity of vasicinone production in our report (6.402% from in vitro leaf) is highest among any other reports on *A. vasica* [(Das et al. 2005 reported 0.047%), (Avula et al. 2008 reported 0.97%), (Suthar et al. 2009 reported 0.45%), (Roja et al. 2011 reported 5.2%), (Garg et al. 2016 reported 0.56%)]. Such augmented production of vasicinone from in vitro leaves might be attributed to the controlled physical environment of growth room and interplay among endogenous and exogenous plant growth regulators during tissue culture.

In vitro culture of plant cell, tissue and organ are accounted as an improved system to yield valuable phytochemicals with medicinal properties (Gantait and Kundu 2017). Plant growth regulators used in vitro was found to augment some metabolic activities and ameliorate phytochemical accumulation in tissue cultured plantlets unlike in their in vivo counterparts (Pandey et al. 2016). According to Gantait et al. (2014), the use of sterilizing agents, plant source, plant growth regulators, culture conditions as well as accumulation of phenolics may significantly enhance the quality of in vitro-regenerated plants. Plants are the main source to provide novel products and act as chemical models for new drug discoveries (Cox and Balick 1994). The rapid development of plant tissue culture and cell culture leads to the production of more secondary metabolites on a large scale (Ramachandra Rao and Ravishankar 2002; Debnath et al. 2006; Gantait et al. 2011). Generally, secondary plant products can be produced throughout the year with the advent of plant tissue culture. Simultaneously, the product would be reliable, predictable and independent of seasonal barriers, plus it complements the conventional production system of secondary metabolites. At times, the in vitro yield of secondary product may exceed from that of the in vivo parent plant parts (Karuppusamy 2009), similar to the present study, wherein the in vitro yield of vasicinone surpassed than that of the in vivo parts of *A. vasica*. Despite the fact that vasicinone can be harvested from any aerial plant part, our findings based on vasicinone profiling revealed that the leaf, especially the in vitro one is the key source. This way of ensuring high vasicinone yield per unit biomass, concurrently through in vivo and in vitro approach, indicates the chemical process of vasicinone to be much easier and more economical.

## Conclusion

Since there is a high demand for vasicinone in the global market of plant-derived drugs, farming activities on *A. vasica* should principally be emphasized on boosting the vasicinone quantity and biomass (especially the leaves). In our present report, we proposed an avenue for large-scale production of vasicinone, simultaneously exploiting both the conventional and tissue culture methods. Furthermore, we substantiated the in vitro clonal propagation as a superior technique to yield the augmented quantity of vasicinone in comparison to in vivo propagation method; the in vitro approach can further be utilized for the amelioration of vasicinone production through biotic and abiotic elicitors and/or stresses developed during tissue culture. The implication of this work can possibly bring down the commercial cost of production of this economically important alkaloid through complementation of in vivo and in vitro approaches.
